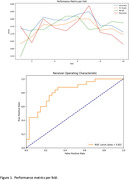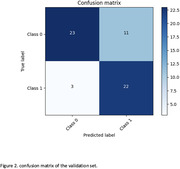# Leveraging T1 MRI Images for Amyloid Status Prediction in Diverse Cognitive Conditions Using Advanced Deep Learning Models

**DOI:** 10.1002/alz.093540

**Published:** 2025-01-09

**Authors:** Seyyed Ali Hosseini, Stijn Servaes, Nesrine Rahmouni, Joseph Therriault, Cécile Tissot, Arthur C. Macedo, Yi‐Ting Wang, Jaime Fernandez Arias, Kely Monica Quispialaya Socualaya, Yansheng Zheng, Tevy Chan, Lydia Trudel, Serge Gauthier, Pedro Rosa‐Neto

**Affiliations:** ^1^ Translational Neuroimaging Laboratory, The McGill University Research Centre for Studies in Aging, Montréal, QC Canada; ^2^ Montreal Neurological Institute, Montreal, QC Canada; ^3^ Douglas Mental Health University Institute, Montréal, QC Canada

## Abstract

**Background:**

Timely and non‐invasive prediction of amyloid status are pivotal in Alzheimer’s disease (AD) diagnostics. This research leverages T1 MRI images to predict amyloid positivity or negativity, offering an economical and less invasive alternative to amyloid PET scans. Using the comprehensive TRAID dataset from McGill University, the study evaluates a spectrum of cognitive conditions including AD, atypical AD, Cognitively Normal (CN), Mild Cognitive Impairment (MCI), MCI not due to AD, Suspected Non‐Alzheimer's Pathophysiology (SNAP), and Vascular MCI (VMCI).

**Method:**

The study involved 588 subjects, representing a cross‐section of cognitive states. A VGG16‐based feature extraction process was meticulously applied to T1 MRI images to capture complex biomarkers of AD. These high‐dimensional features, alongside demographic data, were harnessed to train two deep learning models: a Long Short‐Term Memory (LSTM) model and a Convolutional Neural Network (CNN) ensemble model. To prevent overfitting and data leakage, the models employ 10‐fold StratifiedKFold validation and early stopping, alongside regularization techniques like dropout and batch normalization. Data preprocessing is contained within StratifiedKFolds, and an independent test set is used for unbiased evaluation. The predictive accuracy was scrutinized using a range of metrics, including accuracy, F1‐score, recall, precision, and ROC AUC on the validation sets.

**Result:**

The LSTM model reported average validation metrics with an accuracy of 70.88%, F1‐score of 70.64%, recall of 78.07%, precision of 70.88%, and a ROC AUC of 71.86%. The CNN ensemble model showed a marked improvement, particularly in its recall of 88.00% and ROC AUC of 82.47%, alongside an accuracy of 76.27% and F1‐score of 75.86% (Figure 1 and 2). However, in another model, it was the CNN ensemble model that demonstrated remarkable predictive power, achieving an ROC AUC of 88% within the AD spectrum (CN, MCI, and AD subjects), indicating a high degree of predictive power in classifying amyloid status.

**Conclusion:**

This study underscores the CNN ensemble model's robustness in predicting amyloid status across a broad spectrum of cognitive conditions, with particular emphasis on its application in high‐sensitivity clinical settings. These findings represent a significant advancement in the field of AD diagnostics, contributing to the development of more accessible and cost‐effective prognostic techniques.